# Physicochemical Attributes, Aroma Profile, and Sensory Quality of Organic Crimson Crisp Apples after Storage

**DOI:** 10.3390/foods12091876

**Published:** 2023-05-02

**Authors:** Niklas Pontesegger, Thomas Rühmer, Barbara Siegmund

**Affiliations:** 1 Institute of Analytical Chemistry and Food Chemistry, Graz University of Technology, Stremayrgasse 9/II, A-8010 Graz, Austria; 2 Research Centre for Fruit Growing & Viticulture Haidegg, Ragnitzstraße 193, A-8047 Graz, Austria

**Keywords:** crimson crisp apples, storage, flavor profile, sensory analysis, HS-SPME-GCMS

## Abstract

The objective of this study was to determine the effect of various storage conditions (i.e., storage under regular air with temperature control, controlled atmosphere storage and storage after the application of the ethylene blocker (1-MCP)) on the flavor characteristics of Crimson Crisp apples (*Malus domestica* Borkh.), a scab-resistant cultivar suitable for organic agriculture. Highly ripe organically-grown apples (starch degradation 9.7) were stored under different conditions and evaluated for physicochemical attributes such as fruit weight, firmness, juiciness, total soluble solids, titratable acidity, pH value and aroma profile. The analysis of primary and secondary aroma compounds was conducted utilizing HS-SPME-GCMS and the results were integrated with sensory evaluation. Crimson Crisp apples stored under controlled atmosphere with (MCP) or without (CA) application of 1-MCP, had a higher overall quality than those stored in air (RA) after a storage period of six months. The results from sensory analysis indicated that storage with temperature control alone was not suitable for preserving the distinct properties such as crispness or juiciness of Crimson Crisp apples. However, a significant increase particularly in secondary aroma compounds in RA-stored apples was found accompanied by structural disorders of the apple tissue. While a significant decline in the volatile fraction of CA and MCP-stored apples was observed, PCA showed close correlation between the CA stored and the fresh apple samples. Furthermore, these results demonstrate that the applied measures which are taken to prolong the storage time of Crimson Crisp apples, significantly impact the biochemical reactions in the fruits which are responsible for formation of flavor. These findings underscore the significance of comprehensive aroma analysis for new agricultural products and emphasize the potential for improved the quality of organic apples through carefully considered harvest and storage regimes.

## 1. Introduction

Apples (*Malus domestica* Borkh.) are a seasonal fruit and, in the northern hemisphere, they are typically harvested from late summer to end of autumn. With the technological advancements of the past 100 years, it is now normal for both apple producers and consumers to have good quality apples available throughout the year. The main goal of any storage technique is to slow down fruit metabolism and delay loss of quality and senescence.

Slowing down fruit metabolism significantly impacts biochemical reactions in the fruit. Depending on the fruits’ response to the taken measures, the implication on the fruit quality will be different. The flavor of fruits represents an essential quality criterion as it is directly perceived by the consumer upon consumption. The perception of flavor is a multisensory experience including the perception of taste, texture as well as the ortho- and retronasal odor perception [1]. When investigating the impact of different storage techniques on apples, different aspects of fruit quality need to be considered including physicochemical parameters such as texture, juiciness, sugar concentrations and acidity. Furthermore, it is necessary to investigate the aroma of the produce. Fruit volatiles are basically divided into two groups: (i) primary aroma compounds that are formed and emitted by the intact fruit during maturation and ripening and (ii) secondary aroma compounds that are formed after injury of the fruit (e.g., in course of peeling, cutting or pressing). The formation of the latter groups is based on release of enzymes from the injured cells followed by enzymatically catalyzed reactions in the presence of atmospheric oxygen [2,3]. The presence and the respective enzyme activity and, thus, the formation of primary and secondary apple volatiles is strongly dependent on the apple cultivar as well as on the maturity/ripeness of the fruit [4,5]. The investigation of the fruit volatiles does not only give information on the impact of the storage condition on the flavor formation, but allows us to draw indirect conclusions on how the technical measures influence the biochemical reactions in the apple.

Over the years, a range of different storage techniques have been developed, many of them relying on similar mechanisms [6]. In general, refrigerated storage under regular atmosphere (RA) is the easiest and least expensive method to handle fresh fruits and vegetables. Depending on the sensitivity of each cultivar, apples can be stored between 0–4 °C for periods up to 6 months [7]. The storage period can sometimes be prolonged to 12 months by close monitoring of the produce. The lowered temperatures slow down biochemical degradation and maintain apple quality over a certain period of time [8]. Another well-established storage technique is the so-called controlled atmosphere storage (CA) to extend apple shelf-life while maintaining quality. In addition to low temperatures (−1–6 °C), the surrounding atmosphere of the stored product is altered and controlled. While oxygen concentrations are kept low (2–3%), carbon dioxide levels are increased (1.5–5%) and relative humidity is also kept at high levels (>92%) [9]. Ethylene production is reduced, the respiration rate is decelerated and the enzymatic oxidation rates decrease due to the lower availability of oxygen [10,11]. Moreover, color changes and chlorophyll degradation are reduced while fruit firmness, sugars, organic acids and vitamin concentrations are better maintained [12]. Furthermore, storage disorders such as superficial scald are reduced while senescence is delayed [13]. However, long-term storage under air or controlled atmosphere may lead to fruit disorders and is described not only to be a function of the storage conditions but also the cultivar and the maturity of the fruits [14,15]. Another technique used to delay fruit senescence is the use of ethylene-receptor inhibitors such as 1-methylcyclopropene (1-MCP) [16]. Since this method directly influences the biochemical processes within the fruit, its effect is even more clearly expressed than that of CA storage. 1-MCP dramatically decreases softening and fruit ripening due to a slowdown in respiration [17]. Additionally, the loss of titratable acidity (TA) is delayed and the total volatile content is reduced.

It is described in the literature that different apple cultivars respond in different ways to the measures taken in CA and MCP storage [18]. For example, cultivars such as Gala and Fuji were found to be highly susceptible to carbon dioxide injury in controlled atmosphere storage [19,20], while other cultivars like Golden Delicious are more tolerant to high levels of carbon dioxide [21]. Similarly, cultivars such as Honeycrisp and Cripps Pink are known to be sensitive to MCP treatment, which can cause physiological disorders in the fruit and reduce their storage life [14,22,23]. On the other hand, cultivars like Golden Delicious and Red Delicious were found to be less sensitive to MCP treatment and can tolerate higher 1-MCP concentrations [24,25]. As a consequence, it is important to consider the cultivar-specific responses to different storage methods to maximize the shelf life of apples and avoid any negative impact on the fruits [26].

The quality of the stored product is also dependent on another important aspect: fruit maturity and ripeness [9]. Fruits harvested at different stages of maturity/ripeness perform differently during storage [27]. The general consensus is that fruits intended for storage should be harvested when they are mature but not fully ripe [28]. When this so-called storage ripeness is reached highly depends on the cultivar [29]. This should not only provide optimum organoleptic qualities after storage, but also minimize weight loss occurring during respiratory processes [30]. Apples which are harvested at higher ripeness are more likely to suffer from fruit damage and quality loss because of physiological processes within the fruit, which are summarized by the term senescence [31].

Crimson Crisp is an apple cultivar that was bred and evaluated in the United States by the end of the last century [32]. This cultivar is of high interest for apple breeders in the South of Austria, as it is scab-resistant and does, as a consequence, not require the application of plant protectants. With a high proportion of organic apple farming in this region, there is high potential for scab-resistant cultivars in this region.

Our recent investigations performed on the scab-resistant apple variety Crimson Crisp demonstrated, that this variety is best harvested after a prolonged on-tree ripening period [33]. We showed that the overall quality of the fruit increases significantly when on-tree maturation is prolonged until close to an overripe stage, arguably high starch value (9.7) compared to other cultivars [33]. However, as the suitability for storage is related to the ripeness of apple fruits, there is a need to investigate the storage behavior of late-harvested fruits. In order to understand the impact of long-term storage of highly mature Crimson Crisp apples, we harvested at 166 days after full blossom (dafb) showing a very high starch value of 9.7 and stored them for six months under different storage conditions (RA, CA and MCP). However, the response to the storage conditions depends on the specific apple variety; the predictability is low [14,29,34]. If the highly ripe fruits of high organoleptic quality were not suitable for storage, the prolonged on-tree ripening would not be economically feasible. To close this gap in knowledge, we investigated the storage behavior of Crimson Crisp apples after prolonged on-tree maturation and ripening with respect to their physicochemical attributes and also the development of volatile and potentially aroma-active compounds in combination with their sensory properties.

## 2. Materials and Methods

### 2.1. Plant Material and Storage Techniques

#### 2.1.1. Plant Material

Crimson Crisp apples grafted on M9 rootstocks were cultivated in orchards at the Research Centre for Fruit Growing & Viticulture, Graz-Haidegg, Austria. The apple cultivation was performed following the guidelines for organic farming (AT-BIO-401 according to EN ISO/IEC 17065:2012). Fruits of high ripeness (starch degradation 9.7) were harvested 166 days after full blossom (6 October 2020). The fresh reference sample was analyzed directly after harvest. Due to logistic reasons, the sample preparation for the analysis of primary and secondary volatiles (Section 2.3.1 and Section 2.3.2 respectively) was carried out after keeping the apples at 18 °C overnight. For the investigation of the storage capability of Crimson Crisp apples, the fruits of high ripeness were stored under three different storage regimes (Section 2.1.2).

An intermediate evaluation of a representative sample set was carried out after 126 days in storage (corresponding to four months, ‘4M’) to follow the fruit quality attributes (Section 2.2). The comprehensive analysis (i.e., quality attributes, volatile and sensory analysis) of the fruits was performed after 199 days (corresponding to a storage time of six months, ‘6M’). After the apples had been taken out of the storage facilities, they were allowed to ripen under simulated market conditions at 19 ± 1 °C for 7 days until further processing. The stored fruit samples were compared to fresh (reference) apples of high ripeness (166 dafb), which were analyzed after the harvest.

#### 2.1.2. Storage Techniques

For regular air (RA) storage, the fruits were stored at 2 °C at a relative humidity of 92%. Both temperature and relative humidity were monitored via an automated cooling system (Agri-Datalog, Triggiano, Italy).

In controlled atmosphere (CA) storage, fruits were stored at 2 °C, controlled atmosphere conditions were applied after 1 week, where both oxygen (O_2_) and carbon dioxide (CO_2_) concentrations were adjusted and maintained at 1.5% using CO_2_-scavengers and a N_2_-outlet (Agri-Datalog, Triggiano, Italy). The temperature was monitored via the Agri-Datalog system and cooling was performed by brine as coolant in a compressor system. The relative humidity was generated and monitored via an automatic air humidifier system at 92% (Agri-Datalog, Triggiano, Italy).

The third storage technique (MCP, controlled atmosphere storage with SmartFresh™ application, AgroFresh, Frankfurt/Main, Germany) used a controlled atmosphere equivalent to CA conditions with the additional one-time application of 625 ppb 1-methylcyclopropene (corresponding to 1 g/m^3^) at 18 °C for 24 h before lowering the temperature in the storage facility.

### 2.2. Analysis of Fruit Quality Attributes

#### 2.2.1. Analysis of Fruit Weight, Total Soluble Solids, Fruit Firmness and Juice Content as Well as Browning

For each sample set, 15 fruits were analyzed with the Pimprenelle II (Art. 27050, UP Umweltanalytische Produkte GmbH, Ibbenbüren, Germany), which is a fully automated system to analyze the basic apple quality attributes. In a first step, the individual fruits were weighed on the integrated scale (d = 1 g), final results were given in g. In the second step, the fruit firmness (FF) was determined with a built-in digital penetrometer (d = 15 g), values were given in kg/cm^2^. In the further process, the fruits were crushed to generate apple juice. The juice from each individual fruit was evaluated with a refractometer (±0.2 °Brix) to measure the total soluble solids (TSS), values were given in °Brix. To establish the juice content (JC) of the sample set, the weight of the collected juice was compared to the total weight of the intact fruits and the ratio was given in percent (%).

Browning of flesh and the core was determined by visual assessment of the fruits after being taken out from the storage facility before being analyzed. Only healthy fruits were used for further analysis.

#### 2.2.2. Titratable Acidity (TA)

Potentiometric titration of the total acids quantities was performed using a commercially available auto-titration system (785 DMP Titrino, Metrohm, Herisau, Switzerland). A total of 20 mL of the freshly produced apple juice (see Section 2.3.2) was titrated against 0.1 M NaOH (Convol NORMADOSE, VWR Chemicals, Vienna, Austria) until pH = 10 was reached [33]. The pH value was established at the beginning of each measurement with the integrated pH electrode and the equivalent point of the titration was calculated directly by the titration unit in terms of titration volume (mL). The titratable acidity was calculated from the titration volume at the equivalence point and given in malic acid equivalents.

#### 2.2.3. Starch Degradation

Starch degradation was measured from 15 apples after harvest, which were cut into halves orthogonally to the apple core. The freshly cut site was dried with a paper towel and dipped into an iodine/potassium iodate solution (Lugol’s solution, 3.3 g/L iodine and 6.7 g/L potassium iodide; Carl Roth, Karlsruhe, Germany) and allowed to rest for 5 min. The color intensity of the blue starch-iodine complex represents the degree of ripening in terms of the apple starch degradation. The coloration pattern was matched with the corresponding degradation grade on a 1–10 point scale (EUROFRU, Ctifl—Centre Technique Interprofessionel des Fruits et Legumes, Paris, France) [17], where grade 1 corresponds to no starch degradation and grade 10 to complete starch degradation in the apple fruits. The mean was calculated from 15 apple halves.

### 2.3. Analysis of the Fruit Volatiles

#### 2.3.1. Sample Preparation for the Analysis of Primary Aroma Compounds

Inactivation of apple native enzymes must be achieved as quickly as possible for the analysis of primary aroma compounds to prevent the formation of secondary aroma compounds. To obtain a representative sample, 8 to 10 fruits were peeled and cut into pieces. Immediately after cutting and peeling, an aliquot of approx. a total of 75 g flesh was inserted into a solution prepared from 75 mL deionized water, 30 g NaCl (≥99.5%, Merck, Darmstadt, Germany), 250 mg citric acid monohydrate (puriss., Sigma-Aldrich, Taufkirchen, Germany) and 250 mg L-ascorbic acid (≥99.8%, Sigma-Aldrich, Taufkirchen, Germany) as described by Aprea et al. [35]. The mixture was homogenized in a commercial blender with a glass container to obtain purée-like samples and aliquots of this mixture were immediately deep frozen in glass vials and stored at −25 °C until further use.

#### 2.3.2. Sample Preparation for the Analysis of Secondary Aroma Compounds

Apple juice was prepared from approx. Freshly cut (500 g) and peeled apple pieces from 8–10 fruits with a commercial fruit juicer (centrifuge; Philips Austria, Vienna, Austria). After the samples were let to rest for 30 min in closed glass vials, aliquots were deep frozen at −25 °C until further use.

#### 2.3.3. Analysis of the Apple Volatiles by HS-SPME-GC-MS

For the analysis of primary aroma compounds, aliquots of the homogenized samples (250 mg purée) were transferred into 20 mL headspace vials. 2-Octanol (purity > 99%, Sigma Aldrich, Darmstadt, Germany) was used as internal standard (30 ng absolute in 10 µL methanol corresponding to 120 µg/kg purée, purity > 99%, Fisher Scientific, Schwerte, Germany). For the analysis of secondary aroma compounds, 100 µL of apple fruit juice was used with the addition of 100 mg NaCl and 2-octanol (30 ng absolute in 10 µL methanol corresponding to 300 µg/L juice, purity > 99%, Fisher Scientific, Schwerte, Germany) in 20 mL headspace vials [33]. All samples were analyzed in duplicate in randomized order. The extraction/enrichment of the volatiles was performed by headspace solid-phase micro extraction (HS-SPME) using a CTC Combi PAL sampler (CTC Analytics, Zwingen, Switzerland). A 50/30 µm DVB/Car/PDMS 2 cm stable flex SPME fibre (Supelco, Bellefonte, PA, USA) was used for the enrichment of the volatiles. Prior to the extraction of the volatiles, the samples were equilibrated in the oven of the autosampler at 40 °C for 5 min and the fibre was exposed in the headspace to the equilibrated sample at 40 °C for 20 min. Samples were stirred thoroughly during the equilibration and enrichment process in the oven of the CTC Combi PAL sampler adding a glass-coated stirrer into the headspace vial. Immediately after exposure, the fibre was transferred to the injector of the GC system for thermo-desorption. The SPME fibre was left in the injection port for re-conditioning (20 min) before it was exposed into the headspace of the next sample. Analysis of the volatiles was performed with 1-dimensional gas chromatography-mass spectrometry (GC-MS) (Shimadzu GCMS-QP 2010 Plus, Shimadzu Europa GmbH) on a semi-polar column (Rxi^®^ 5 ms, 30 m × 0.25 mm × 1 µm, Restek Corporation, Bellefonte, PA, USA). The following conditions were used: temperature program starting at −10 °C for 1 min with a temperature ramp of 8 °C/min up to 270 °C (1 min holding time), splitless injection with a sampling time of 1 min and an injection temperature of 270 °C was applied. SPME liners with a constant inner diameter of 0.75 mm were used in the GC injection system and cryo-focusing was performed by blowing liquid nitrogen into the GC oven to reach the start temperature of −10 °C. Helium was used as carrier gas with a linear velocity of 35.0 cm/s. Mass-selective detection was performed in the scan mode (35–350 *m*/*z*, EI (70 eV), interface temperature 280 °C, ion source temperature 200 °C).

The obtained chromatograms were analyzed with the free deconvolution software PARADISe [36], which is based on the PARAFAC2 model [37]. The identification of the compounds was performed on probability-based matching of the mass spectra to those from MS libraries (NIST14, Adams Essential Oils library, FFNSC 4.0) or authentic reference compounds as well as on linear-temperature programmed retention indices (RI) [38]. RI was calculated using the homologous series of n-alkanes (C5–C26) and was compared to RI from authentic reference compounds or data from the literature. The relative concentrations (semi-quantification) were calculated by comparison of the respective peak areas obtained from the total ion chromatograms deconvolution to the internal standard 2-octanol assuming a response factor of 1 for all compounds as described by Elmore (2015) [39]. Relative concentrations of primary aroma compounds were calculated from the sample quantity taking the relative fruit content of the purée into account and are given in mg/kg. Relative concentrations of secondary aroma compounds were calculated directly from the juice and are given in mg/L juice. To follow changes within compound class (alcohols, esters, etc.), the relative amounts of all compounds of one family were summed up.

### 2.4. Sensory Analysis

#### 2.4.1. Panel Training and Selection

Sensory analysis was performed by a well-trained panel under standardized conditions in the sensory laboratory. All panelists (eight females and three males aged between 25 and 57 years) fulfilled the basic requirements given by DIN EN ISO 8586 and all but two had several years of experience conducting sensory evaluations. Additionally, training sessions specific to apple flavor were carried out prior to the study. This training sensitizes the panelists to the expected sensory impressions during apple analysis. On the one hand, the training focused on volatiles as well as taste stimuli present in apples. For the aroma training, solutions of relevant compounds were prepared in ethanol in adequate concentrations (1–2%). Filter strips were dipped into these solutions, dried and stored in cellophane coats until sensory evaluation. The following compounds were used for the odor training: ethyl decanoate, α-ionone, α-terpineol, linalool, 5-hexenyl acetate, hexyl butanoate, 2-hexenal (*E*), 3-hexenol (*Z*), ethyl butanoate, β-damascenone. At the time of the investigation, all compounds were registered in the European Union as flavoring compounds and authorized to be used in flavored foods according to regulation (EU) No 872/2012. The compounds were purchased from Sigma-Aldrich (Vienna, Austria) and had a purity of ≥96% (food grade quality). Apple juice samples with adjusted sweetness and sourness were prepared to train the taste evaluation of apple fruits. The panelists were asked to rank four apple juice samples according to their increasing sensation of sweetness (sucrose addition: 1.: +1 g/L, 2.: +5 g/L, 3.: +10 g/L, 4.: +15 g/L) and acidity (citric acid addition: 1.: +0.1 g/L, 2.: +0.5 g/L, 3.: +1.0 g/L, 4.: +1.5 g/L), respectively. Furthermore, apples of different varieties, origins and degrees of ripeness were presented to the panelists in two sessions for sensory evaluation. The results of the versatile evaluations were discussed among the panel members.

#### 2.4.2. Sample Preparation for Sensory Evaluation

Intact apples were washed and cut into 12 pieces each. To avoid the browning of the surface of the apple pieces during the time between sample preparation and sensory evaluation, we followed the protocol published by Corollaro et al., 2013 [40]. To achieve this, the stem, core and seeds were removed and the apple slices were dipped into an antioxidant solution (0.2% citric acid, 0.2% ascorbic acid, 0.5% calcium chloride; all 98%, Darmstadt, Merck) for 30 s. A minimum of three apple slices from three different apples was offered to each panelist in a sealed cup with a randomized 3-digit code for each sample.

#### 2.4.3. Sensory Evaluation the Stored Apples Using Open-Ended Questioning (OEQ)

The effect of the different storage conditions on the sensory properties of Crimson Crisp apples was not predictable. We thus decided to give preference to open-ended questioning OEQ [41]. OEQ was primarily designed for the use with consumers, but is also well suited for use with a lower number of trained panelists [42]. The panelists were asked to evaluate freshly cut apples from the RA, CA and MCP storage experiments. Each panelist received a full sample set labeled with random 3-digit number codes and the samples were evaluated in random order. The panelists were asked to describe aroma, texture and taste/flavor as detailed as possible. The results were collected manually and similar terms were combined to one term (e.g., ‘green’ and ‘green/grassy’ are summarized as ‘green’). Descriptors that were named fewer than three times in total and that could not be associated with any other descriptor were eliminated.

### 2.5. Statistical Data Treatment

The statistical analysis of the data was performed with the MS Excel add-in XLSTAT (Addinsoft (2021), XLSTAT statistical and data analysis solution. Long Island, NY, USA). For the identification of significant concentration differences between the samples and their storage conditions, one-way analysis of variance (ANOVA) was performed (*p* = 0.05). If statistically significant differences were observed within the data sets, the ANOVA was followed by post-hoc pairwise comparison using Tukey’s Honestly Significant Difference (HSD) test control (*p* < 0.05) to check for differences between single samples. Correlations between samples and volatiles were analyzed by principal component analysis (PCA) using Pearson correlation.

Statistical treatment of the OEQ-data was performed from the resulting contingency table via correspondence analysis based on Chi-square statistics (*p* < 0,05) (MS Excel add-in XLSTAT (Addinsoft (2021), XLSTAT statistical and data analysis solution. Long Island, NY, USA).

## 3. Results and Discussion

### 3.1. Quality Attributes of Stored Apples

The highly ripe fresh Crimson Crisp apple, harvested 166 dafb, is a bright red fruit [32] with an average fruit weight of 189 ± 26 g. With a total soluble solids (TSS) value of 13.9 ± 0.3 °Brix and starch degradation of 9.7, the sample showed relatively high values compared to other cultivars [43]. The fruit firmness (FF) of Crimson apples at this stage of high ripeness is higher compared to other cultivars with an average value of 7.9 ± 0.9 kg/cm^2^ [44]. The titratable acidity (TA) and the juice content (JC) were comparable to those of other cultivars at optimum harvest conditions [43].

Quality attributes were evaluated and compared to the fresh apples after an intermediate storage control of 126 days in storage (4M) and after 199 days in storage (6M) (Table 1). The fruit weight changed mainly due to a loss of water in fruit through transpiration. However, the rate of transpiration is mostly dependent on the surrounding atmosphere of the product, this is described as a pure physical effect [45]. We observed that Crimson Crisp apples from RA, CA or MCP storage did not show significant differences compared to the fresh fruit nor between the sample sets, respectively. Also, the prolonged storage of six months compared to four months did not lead to a significant change in fruit weight.

The TSS showed a significant difference between apples stored in RA and in other storage conditions already after four months of storage. After six months, fruits stored under RA conditions (6M-RA) showed significantly lower TSS values compared to the fresh apples after harvest. On the contrary, 6M-CA and 6M-MCP did not show significant differences in TSS to fresh fruits. Small changes in the sugar content, which is directly correlated to the TSS content, upon storage were also reported in previous studies [46]. However, it is noteworthy that, in general, TSS show minor, for CA and MCP storage not statistically significant, decrease in Crimson Crisp apples during storage, while it reportedly increases during storage of other cultivars, among them the well-known varieties Golden Delicious and Pink Lady [47,48].

A decrease in fruit firmness can generally be expected during storage of apples [49]; however, the impact is reportedly cultivar dependent [50]. This makes the evaluation for each cultivar necessary as the firmness is considered a good parameter to predict mealiness in apples [51]. We observed a strong decline in FF after four and six months for RA storage. Values of 6.1 and 6.2 kg/cm^2^ were reached, which is a significant difference to both the fresh samples and fruits stored under controlled atmosphere. No statistically significant differences in firmness were observed for Crimson Crisp apples neither stored under CA atmosphere nor under CA conditions with 1-MCP application. The observed, although not significant, changes in MCP storage are most probably due the natural variability of the fruits.

Titratable acidity reportedly decreases during storage of apples which, in general, has strong impact on the sensory properties of the fruits [52]. This behavior was also observed for stored Crimson Crisp apples. After six months of storage time, in RA-stored apples TA decreased to only approximately 50% compared to fresh apples, reaching a value of only 8.8 g malic acid equiv./L. In comparison to RA apples, CA and MCP apples showed smaller decreases in acidity over the observed storage period. TA loss in CA-stored fruits was approximately 40%, while MCP-stored fruits still contained higher amounts of organic acids (~20% loss). The observed small decrease in TA of MCP-stored apples corresponded to the expectations, since the conversion of organic acids to other products is mainly associated with higher respiration rates [53].

The juice content (JC) was analyzed to evaluate possible effects on further processing of this cultivar. Furthermore, the JC also reflects the juiciness of the fruits which is an important sensory aspect for consumers upon consumption. We observed a high decrease of JC after four and six months of storage in RA facilities. The juice yield dropped by almost 50% under RA storage. Interestingly, storage longer than four months did not further decrease the JC value in RA-stored fruits. Compared to the fresh apples, samples from CA and MCP storage only showed differences of <10% in the juice content. This behavior might indicate disorders in RA apples leading to JC loss, even though the fruit weight did not show significant difference.

Browning of the flesh and the core housing represents a fruit characteristic that is directly perceived by the consumers and is, thus, of high relevance, even though internal browning disorders are not yet completely understood [29,54]. The results of this study demonstrate that Crimson Crisp apples are not prone to browning of the apple tissue and the core when stored under a controlled atmosphere. When stored under RA conditions, no browning was observed after four months of storage. However, approximately one-quarter of RA stored apples were affected after a period of six months.

### 3.2. Volatilome of Apples after Storage

Volatile compounds are of high importance for the odor of apples. In addition to texture, acidity and sweetness, the behavior of volatile compounds is a measure for the biochemical changes occurring in the fruit upon storage as the volatilome of the stored apples is affected by the storage conditions [53]. The general reduction of volatiles during controlled atmosphere storage—with or without MCP application—was described previously [55]. The formation of volatiles is determined by the activity of several enzyme systems that are more or less affected by the reduction of fruit respiration and ethylene biosynthesis. A previous study also reported different post-storage recovery rates of the fruits with respect to their biochemical activities showing higher aroma compound formation for RA storage than for CA or MCP conditions [56].

**Figure 1:**
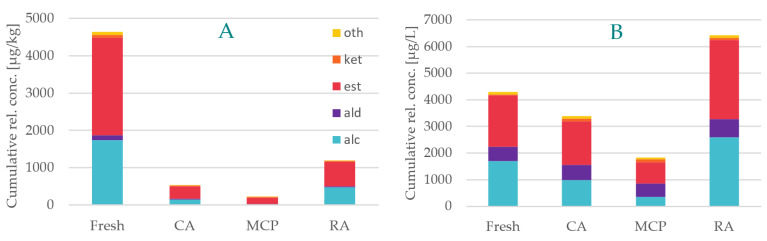
Volatilome in stored Crimson Crisp apples. Cumulative relative concentrations of primary (**A**) and secondary (**B**) volatiles in the fresh apples after harvest (Fresh) compared to stored apples from regular air storage (RA), controlled atmosphere storage (CA) and controlled atmosphere storage with 1-MCP application (MCP). (alc = alcohols; ald = aldehydes; est = esters; ket = ketones, oth = others).

In this study, the volatilome of the highly ripe apples was analyzed after six months of postharvest storage under different storage conditions and compared to those of the same apples that were analyzed immediately after the harvest. A total of 52 compounds were identified as primary aroma compounds (i.e., 13 alcohols, six aldehydes, 22 esters, four ketones and seven other compounds) and 50 different compounds as secondary aroma compounds (i.e., 12 alcohols, six aldehydes, 22 esters, four ketones and six other compounds). A graphic representation of the cumulative relative concentrations of different compound classes and storage techniques is given in Figure 1. The detailed results on the semi-quantitative data of the fruit volatiles (primary and secondary flavor compounds) are given in Table 2.

In general, the different storage techniques slow down the reaction rates of biochemical reactions in the fruits [10]. In this study, the comparison of the volatilome showed significant differences in the behavior of primary and secondary aroma compounds after postharvest storage compared to the apples analyzed immediately after harvest, but also relevant differences that are dependent on the type of storage. The fresh apples arguably exhibit similar levels of both primary and secondary aroma volatiles (more than 4000 mg/kg fruit or mg/L juice respectively); however, after storage in general, we observed noticeably lower amounts of primary aroma volatiles.

The extraordinarily high loss of primary aroma compounds was observed when comparing the volatiles of the stored fruits with those of apples analyzed immediately after the harvest at 166 dafb. An overall loss of more than 70% of primary aroma compounds was observed after RA storage with even higher losses in the total volatiles for apples from CA and MCP storage (Figure 1A). Strikingly, the fraction of alcohols as part of the primary volatilome of MCP-stored apples decreased by 99%. It is highly plausible that these changes in the volatilome affect the sensory sensation for consumers. As for the TSS behavior (see Section 3.1), the results given in Figure 1A indicate that the respiration rate and, thus, the bioflavor formation rates are reduced in the order RA > CA > MCP. This is in accordance with data from previous papers. For apples of the varieties other than Crimson Crisp, strong reduction of the biosynthesis of volatiles had been reported, especially after MCP treatment [55,57].

It is interesting to note that in Crimson Crisp apples, we observed a general reduction of the primary aroma compounds in the stored fruits, whereas for secondary aroma compounds we observed significantly lower amounts of volatiles in juices produced from CA storage with or without MCP application, but increased amounts in the juice produced from apples stored under regular atmosphere. In general, secondary aroma compounds are formed after cell disruption and release of enzymes that are genuine to apple cells. The formation of volatiles is on the one hand depending on the activity of the enzymes but on the other hand on the availability of the precursors that are in most cases not odor-active.

Based on the results obtained from the analysis of the browning and the juice content, we assume that physiological disorders take place in RA apples after a six months storage period (Section 3.1). The observed discrepancy between primary and secondary aroma compounds in RA-stored Crimson Crisp apples suggests the conclusion that these physiological disorders might make non-volatile precursors better available for the formation of secondary aroma compounds. However, this assumption requires future investigation.

The major trend throughout all compound classes, particularly of alcohols and esters, is the presence of highest amounts of volatiles after RA storage and the lowest amounts of volatiles after MCP storage. The only exception is ketones, which show a contrary behavior. This behavior of increasing ketone concentrations compared to fresh fruits corresponds to previous observations made from Golden Delicious and Delicious apples after MCP storage over a period of one month [58]. However, although ketones show a general three-to-four-fold increase in CA and MCP storage, their impact on the overall flavor is expected to be negligible as their concentrations are low. Aldehydes in general are less affected by the storage conditions; their relative concentrations stayed more or less constant with varying relative amounts between 96 and 127% in secondary volatiles. Again, RA storage showed the highest amounts of aldehydes. Aldehydes are final reaction products of fatty acid degradation following the lipoxygenase (LOX) pathway. A reduced LOX activity as the reason for lowered aldehyde concentrations in Galaxy apples was reported in the literatures [55].

A mixed behavior in dependence of the storage conditions was observed for alcohols. While we observed a general decrease in the overall alcohol concentrations in the order of fresh fruits > RA > CA > MCP for primary aroma compounds, we could observe a reduction of the overall quantities of alcohols in secondary aroma compounds from fresh fruits to CA storage by 42% and 80% in MCP storage, while the total amount of alcohols as part of the secondary aroma compounds increased by 53% in RA storage. Enzyme systems that are involved in the formation of straight chain alcohols are lipoxygenase (LOX) and alcohol dehydrogenase (ADH). Several groups reported a reduced activity of the enzyme ACC (1-aminocyclopropane-1-carboxylate) oxidase depending on the storage condition in various apple varieties with MCP application being most effective [57,59,60]. ACC oxidase catalyzes the last step within the ethylene biosynthesis pathway and may be used as a measure for the reduction of the autocatalytic ripening processes. The observed reduction in alcohol concentrations in dependence of the storage conditions in this study in Crimson Crisp apples indicate this reduction in ACC oxidase activity. Our findings which show that alcohols from primary aroma compounds follow the described trend, but not the alcohols determined as part of the secondary aroma compounds again indicate that in addition to the enzyme activities, structural changes occur in Crimson Crisp apples in the course of long-term RA storage making non-volatile precursors better available. It is also noteworthy, that the two branched-chain alcohols 2-methylbutanol and 2-methyl-2-buten-1-ol did not follow the trend that was reported for straight-chain alcohols. No clear trend in concentrations can be seen for these compounds in relation to the storage conditions. In contrast to straight-chain alcohols, which are degradation products from fatty acids, branched-chain alcohols are formed by the degradation of the corresponding amino acids and the activity of the enzyme branched-chain amino acid transaminase (BCAT). Xiaotang et al., 2016 [61] reported a lower dependence of BCAT activity in Golden Delicious apples on ethylene than for enzymes involved in the fatty acid degradation. The observed alcohol concentrations in Crimson Crisp apples indicate a similar reaction on the reduction of ethylene formation.

Esters are compounds that are of great importance for the flavor of apples. The fruity notes are mainly derived from this compound class [62]. The concentration patterns of esters follow the general trend of the straight chain alcohols. Whereas in primary aroma compounds, we observed a decrease in ester concentrations following fresh apples > RA > CA > MCP, the relative concentration of alcohols as part of the secondary aroma compounds was highest in RA after storage followed by fresh apples > CA > MCP. Esters are results of ester formation from the corresponding alcohols upon activity of the enzyme acyltransferase (AAT). Such as LOX and ADH, AAT activities are ethylene dependent [63,64], thus, their formation is reduced along with the reduction of the respiration rate and the ethylene formation. As a consequence, ester concentrations follow similar patterns as alcohols which may also explain the observed high amounts of esters in juice from RA stored Crimson Crisp apples.

**Figure 2:**
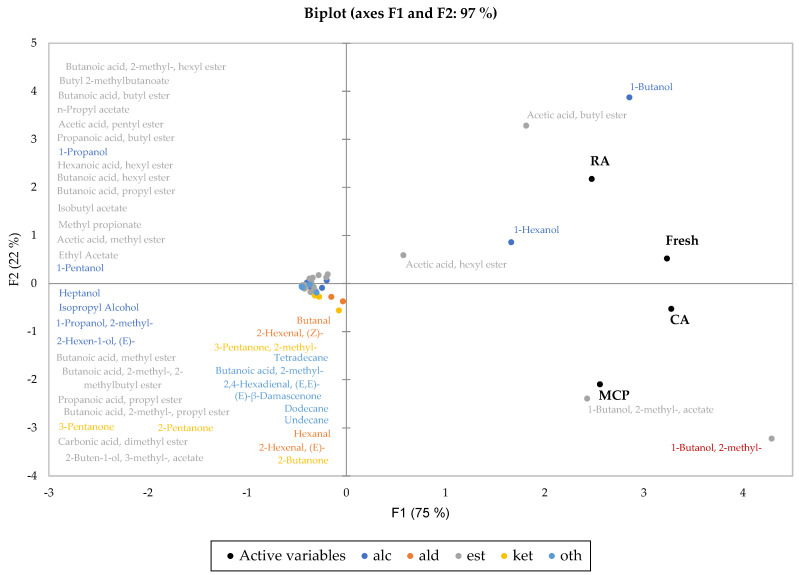
Principal component analysis (PCA, Pearson correlation) biplot of the volatile compounds (primary aroma compounds) of fresh and stored Crimson Crisp apples (variables) and the storage techniques (observables) calculated with the relative concentrations of the volatiles given in Table 2. Compound names given in quadrant II and III appear in the order of their position from top to bottom of the cluster near the centroid of the biplot. (alc = alcohols; ald = aldehydes; est = esters; ket = ketones, oth = others).

Figure 2 shows the biplot as a result of the multivariate data analysis (principal component analysis PCA) calculated from the relative concentrations of the secondary aroma compounds and demonstrates the correlations of the Crimson Crisp apples stored under different conditions and the corresponding volatiles. PCA analysis calculated by means of the primary aroma compounds delivers a similar picture (results not shown). The results show a close relation of fresh apples after the harvest and apples stored under regular air on the one hand (quadrant I) and a correlation of CA and MCP stored Crimson Crisp apples on the other hand (quadrant IV). Even though we observed strong differences in the overall amounts of straight-chain alcohols, esters and aldehydes in dependence of the storage conditions, this is not reflected in the results obtained from PCA analysis. The bigger part of volatiles is located in a dense cluster in quadrant II and III close to the centroid of the PCA plot. Five distinct volatiles may be regarded as discriminative for the storage behavior of Crimson Crisp apples stored under different conditions. While the straight-chain alcohols 1-butanol and 1-hexanol together with the corresponding acetates butyl acetate and hexyl acetate are closely correlated with fresh apples and RA stored apples, the methyl-branched-chain alcohol 2-methyl butanol and its corresponding ester 2-methyl butyl acetate exhibit high correlations with CA and MCP stored fruits. These findings again indicate a strong down-regulation of enzymes by regulation of ethylene emission, while enzymes that are necessary for amino acids degradation are less affected by measures reducing or inhibiting ethylene formation by the fruits. However, CA-stored apples are more related—and probably more similar in their aroma properties—to fresh apples than MCP-stored apples.

The prediction of the sensory properties for these products based on this dataset is scarcely possible. Reduced aroma—in both intensity and complexity—is expected from CA and even more pronounced from MCP apples, while RA samples would most probably show a more intense aroma. However, the results of the analysis of volatiles suggest that CA storage might best preserve the qualities of Crimson Crisp apples that are harvested at high ripeness.

### 3.3. Sensory Analysis of Stored Crimson Crisp Apples

**Figure 3:**
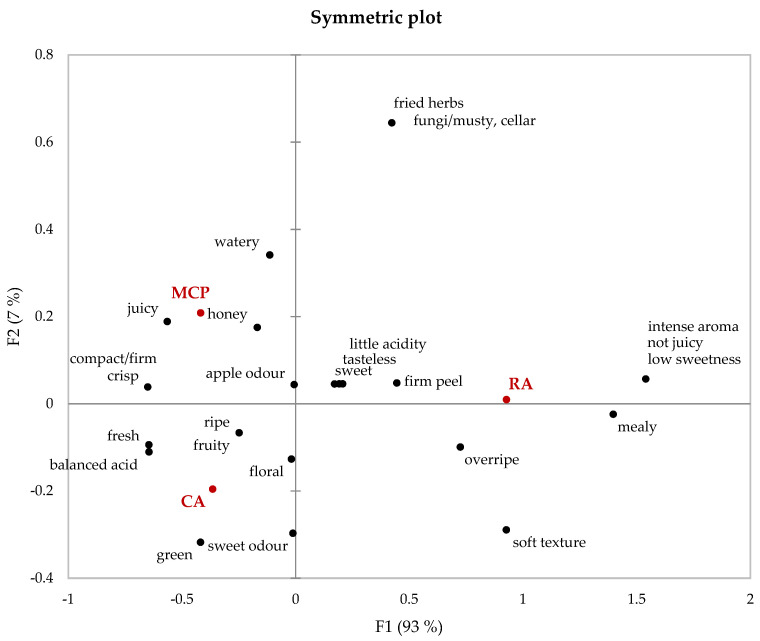
Symmetric plot (axis F1 and F2; 100%) of the correspondence analysis of the open-ended questioning (OEQ) based on Chi-square statistics (*p <* 0.05). RA = regular air storage, CA = controlled air storage, MCP = controlled air + 1-MCP.

The analysis of the fruit volatilome delivers valuable information about the formation and degradation of volatiles, whether or not they are odor-active. However, to understand the impact on the overall flavor of the fruits, sensory evaluation is required. For the sensory analysis of the stored Crimson Crisp fruits, a trained panel was asked to describe the aroma, texture and overall flavor of the three samples RA, CA and MCP by using ‘open-ended questioning’ (OEQ) as the sensory technique [41]. The application of this descriptive technique makes it possible to assess data about the stored apples beyond the scope of the predefined CATA analysis for fresh apples that we applied to follow the fruit development during on-tree ripening [33]. OEQ results were visualized as a biplot as a result via correspondence analysis (Figure 3).

The symmetric plot, which is a 2-dimenionsal representation of the OEQ results, shows a clear differentiation between the RA apples and the apples stored under CA and MCP along the *x*-axis with the principal component F1 explaining 93% of the variability. Furthermore, the results show that RA-stored apples were correlated with a very intense aroma. This is in high accordance with the results obtained from GC-MS analysis of the volatilome (Section 3.2), where compounds such as 1-butanol (medicine, wine, fruit),butyl acetate (pear) and butyl 2-methylbutanoate (fruit, cocoa) showed the highest relative concentrations in the secondary volatilome (odor descriptors [65]). The texture was described as soft, mealy and not juicy. This is in line with our results for firmness, which showed that the firmness and juiciness of RA apples decreased significantly during storage (Section 3.1). The apples were also reported to be overripe and in contrast to the soft pulp, they exhibited a firm apple peel. Both descriptors are relatively less desirable for the choice of table apples [40,66].

The results given in Figure 3 also show that apples stored in CA and MCP were very similar to each other as the principal component F2 only counts for approx. 7% of the variance. The descriptors located in quadrants II and III are closely related to both sample sets from CA and MCP storage. Both samples were correlated to ripe, crisp/compact apples. CA and MCP fruits exhibit a balanced sweet-sour ratio and they were perceived as fresh with apple and fruity odor sensations by the sensory panel. Even though F2 only accounts for 7% of the variance, differences were observed between the two apple samples CA and MCP. The fruits stored in CA storage express a higher fruitiness, with a sweet aroma and also have floral and green notes. Although CA apples had the highest TSS content, this is not reflected in the sensory properties of the fruits. In contrast to CA apples, MCP apples do not show expressed fruitiness. The sensory descriptors are dominated by texture attributes (crispness, juiciness, a ‘watery’ impression). These results correlate well with those obtained from the analytical data—MCP apples showed the lowest concentrations in volatile compounds, whereas the pulp firmness and juiciness were highest among the investigated fruits.

## 4. Conclusions

In this study, we evaluated the applicability of different conditions during long-term storage for highly ripe apples (starch degradation 9.7) of the scab-resistant and organically grown cultivar Crimson Crisp. The results demonstrate that it is necessary to evaluate the ccultivar-specificbehavior in the course of long-term storage. Under CA and MCP storage conditions, Crimson Crisp apples were able to maintain their firmness and juiciness, did not show browning and only exhibited minor changes in TSS, TA and pH. On the contrary, RA-stored fruits suffered from a severe loss of quality, which was especially noticeable in the case of TA, firmness and juiciness.

The analysis of primary and secondary aroma compounds shows a strong impact on the biochemical reactions in the fruit by the taken measures. While the overall content of primary volatile organic compounds was significantly reduced regardless of the storage technique, the highest overall amounts of secondary aroma compounds were found in RA-stored fruit. These results do not only indicate a less pronounced impact of RA storage conditions on enzyme systems which are responsible for the formation of volatiles, but indicate structural disorders in RA stored fruits resulting in a better availability of non-volatile aroma precursors. On the contrast to RA-stored fruits, both CA and MCP-stored fruits showed overall losses in secondary aroma compounds after 6 months, which were even more pronounced in MCP-stored apples than in CA-stored apple. However, multivariate data treatment of the concentrations of the aroma volatiles demonstrated that especially CA stored fruits show the highest correlation to the fresh sample. This leads to the conclusion, that CA-stored apples show reduced flavor but the overall flavor profile is best conserved. These findings are strongly supported by the results obtained from sensory evaluation which confirm the preservation of fresh/fruity attributes as well as crisp and juicy texture in CA and MCP stored fruits, while RA leads to structural disorders in Crimson Crisp apples with concomitant overbearing aroma formation.

Crimson Crisp apples represent an interesting and promising cultivar for this region. The results demonstrate that Crimson Crisp apples of high ripeness are suitable for long-term storage under controlled atmosphere. Even though RA storage was shown as not suitable, CA storage proved to be an appropriate measure. This knowledge gain is of particular importance for the storage of organically grown Crimson Crisp apples, as the application of 1-MCP is not allowed for organic fruits. In addition to the knowledge that we gain for pomology and apple cultivation, this work highlights the power of flavor analysis and sensory evaluation when new cultivars are introduced to orchards and tested for their quality potential.

## Figures and Tables

**Table 1:** Physical and chemical quality attributes of Crimson Crisp apples at harvest and four months (4M) and six months (6M) of storage under different conditions. The results of one-way ANOVA followed by post-hoc pairwise comparison using Tukey’s Honestly Significant Difference (HSD) test control (*p <* 0.05) to check for differences between single samples are reported as letter codes. ^A–D^ Averages with the different letters are significantly different. Browning of the flesh or core, respectively, is given in percent affected fruits.

Samples	Weight [g]	TSS [°Brix]	FF [kg/cm^2^]	TA [g Malic Acid Equiv./L]	pH	Juice Content [%]	Flesh/Corebrowning [%]
Fresh	189 ± 26 ^A^	13.9 ± 0.3 ^C^	7.9 ± 0.9 ^BC^	17.5 ± 0.1 ^D^	3.32 ± 0.01 ^A^	22.5	0/0
4M-RA	192 ± 21 ^A^	12.7 ± 0.1 ^A^	6.2 ± 0.6 ^A^	/	/	12.9	0/0
4M-CA	202 ± 21 ^A^	13.6 ± 0.2 ^BC^	7.8 ± 0.5 ^BC^	/	/	20.6	0/0
4M-MCP	210 ± 30 ^A^	13.6 ± 0.5 ^BC^	7.4 ± 0.7 ^B^	/	/	20.2	0/0
6M-RA	193 ± 23 ^A^	12.8 ± 0.2 ^A^	6.1 ± 0.5 ^A^	8.8 ± 0.3 ^A^	3.98 ± 0.01 ^D^	13.1	27/13
6M-CA	194 ± 33 ^A^	13.3 ± 0.5 ^B^	7.7 ± 0.6 ^BC^	10.7 ± 0.1 ^B^	3.80 ± 0.01 ^C^	21.3	0/0
6M-MCP	190 ± 28 ^A^	13.7 ± 0.5 ^BC^	8.3 ± 0.7 ^C^	14.0 ± 0.1 ^C^	3.68 ± 0.01 ^B^	21.2	0/0

**Table 2:** Mean values (relative concentrations to IS given in µg/L) (n = 2 for Fresh, n = 3 for CA, MCP and RA) of the primary flavor compounds identified in apple purée after inhibition of fruit enzymes and secondary flavor compounds determined in in fruit juice in Crimson Crisp apples before storage (166 dafb) and after 199 days storage under different conditions (CA—controlled atmosphere storage, MCP—controlled atmosphere storage with the application of 1-MCP, RA—regular air storage).

	Primary Aroma Compounds						Secondary Aroma Compounds			
Compound Name ^a^	RI (exp.) ^b^	RI (lit.) ^c^	Fresh ^d^ [µg/kg]	CA ^d^ [µg/kg]	MCP ^d^ [µg/kg]	RA ^d^ [µg/kg]	Fresh ^d^ [µg/L]	CA ^d^ [µg/L]	MCP ^d^ [µg/L]	RA ^d^ [µg/L]
***Alcohols***										
2-Propanol	508	506	1.17 ^A^	0.33 ^B^	0.34 ^B^	0.25 ^B^	0.92 ^B^	0.98 ^B^	1.94 ^A^	1.50 ^AB^
1-Propanol	565	558	22.7 ^A^	0.19 ^C^	0.15 ^C^	6.59 ^C^	23.5 ^B^	3.48 ^C^	0.3 ^C^	44.6 ^A^
1-Propanol, 2-methyl-	632	635	28.2 ^A^	8.81 ^B^	2.12 ^C^	9.85 ^C^	n.d.	n.d.	n.d.	n.d.
1-Butanol	668	670	777 ^A^	75.4 ^C^	2.30 ^D^	364 ^D^	772.89	475 ^BC^	96.9 ^C^	1850 ^A^
1-Butanol, 2-methyl-	740	738	876 ^A^	153.7 ^B^	82.5 ^C^	94.1 ^C^	703 ^B^	825 ^A^	456 ^C^	500 ^C^
1-Pentanol	767	771	121.8 ^A^	4.50 ^C^	0.39 ^D^	6.90 ^D^	121.9 ^A^	30.9 ^B^	3.58 ^C^	40.4 ^B^
2-Buten-1-ol, 2-methyl- ^t^	774	n.a.	n.d. ^D^	17.2 ^A^	6.46 ^B^	1.86 ^C^	10.4 ^C^	108 ^A^	55.1 ^B^	10.2 ^C^
2-Hexen-1-ol, (*E*)-	866	867	23.6 ^A^	2.97 ^B^	1.69 ^C^	2.76 ^C^	2.61 ^A^	27 ^B^	32.0 ^B^	32.0 ^B^
1-Hexanol	869	870	763 ^A^	55.6 ^C^	4.71 ^D^	82.1 ^D^	737 ^A^	323 ^C^	143 ^D^	537 ^B^
Heptanol	968	972	6.55 ^A^	0.58 ^C^	0.21 ^D^	1.63 ^D^	6.74 ^B^	3.83 ^C^	1.57 ^D^	10.6 ^A^
1-Hexanol, 2-ethyl-	1029	1031	n.d. ^D^	0.46 ^C^	0.36 ^C^	0.52 ^C^	3.53 ^AB^	3.32 ^AB^	2.92 ^B^	3.70 ^A^
1-Octanol	1069	1070	n.d. ^D^	0.39 ^C^	0.08 ^C^	1.63 ^C^	12.63 ^A^	0.79 ^C^	0.15 ^C^	6.81 ^B^
1-Decanol	1273	1272	n.d. ^C^	0.24 ^B^	0.15 ^B^	0.40 ^B^	1.01 ^A^	4.14 ^A^	2.8 ^A^	8.3 ^A^
***Aldehydes***										
Butanal	593	598	4.74 ^A^	1.26 ^C^	0.90 ^C^	3.24 ^C^	4.77 ^BC^	6.1 ^B^	4.3 ^C^	13.3 ^A^
Hexanal	799	802	27.4 ^A^	10.8 ^B^	6.18 ^C^	11.5 ^C^	149.5 ^A^	254 ^A^	245 ^A^	357 ^A^
2-Hexenal, (*Z*)-	846	848	3.43 ^A^	0.13 ^B^	0.08 ^B^	0.10 ^B^	10.8 ^A^	12.0 ^A^	9.48 ^A^	10.8 ^A^
2-Hexenal, (*E*)-	855	854	90.5 ^A^	10.85 ^B^	8.42 ^BC^	7.77 ^BC^	357 ^A^	272 ^B^	243 ^B^	282 ^B^
2,4-Hexadienal, (*E,E*)-	912	913	2.31 ^A^	0.43 ^B^	0.34 ^B^	0.41 ^B^	13.3 ^A^	14.1 ^A^	12.8 ^A^	12.6 ^A^
Octanal	1004	1005	n.d. ^D^	2.77 ^B^	1.01 ^C^	2.79 ^B^	3.9 ^B^	8.62 ^A^	3.4 ^B^	11.0 ^A^
Nonanal	1106	1106	n.d. ^C^	3.16 ^B^	2.29 ^B^	3.73 ^B^	12.9 ^A^	9.3 ^A^	10.7 ^A^	11.8
***Esters***										
Acetic acid, methyl ester	535	525	2.66 ^B^	1.48 ^BC^	0.79 ^C^	3.65 ^C^	0.77 ^C^	4.02 ^B^	2.1 ^C^	12.78 ^A^
Acetic acid, ethyl ester	618	620	7.26 ^B^	3.23 ^C^	4.02 ^C^	9.90 ^C^	2.41 ^C^	8.42 ^B^	9.43 ^B^	23.0 ^A^
Carbonic acid, dimethyl ester	621	620	2.35 ^A^	0.74 ^B^	0.76 ^B^	0.61 ^B^	1.72 ^C^	2.69 ^A^	2.94 ^A^	2.18 ^B^
Propanoic acid, methyl ester	634	643	3.54 ^A^	0.95 ^BC^	1.07 ^B^	0.63 ^B^	1.05 ^C^	2.02 ^AB^	2.39 ^A^	1.4 ^BC^
Acetic acid, propyl ester	714	720	45.8 ^A^	0.29 ^C^	0.01 ^C^	13.9 ^C^	25.5 ^B^	4.07 ^C^	0.83 ^C^	48 ^A^
Butanoic acid, methyl ester	724	728	10.9 ^A^	2.93 ^B^	2.97 ^B^	2.54 ^B^	2.8 ^D^	9.7 ^B^	11 ^A^	8.0 ^C^
Acetic acid, 2-methylpropyl ester	774	780	10.68 ^A^	5.01 ^C^	1.68 ^D^	8.31 ^D^	6.77 ^C^	21.2 ^B^	7.75 ^C^	32.4 ^A^
Propanoic acid, propyl ester	809	812	12.1 ^A^	0.01 ^C^	n.d. ^C^	0.69 ^C^	8.51 ^A^	0.64 ^C^	0.05 ^D^	3.95 ^B^
Acetic acid, butyl ester	813	817	536 ^A^	38.1 ^C^	0.73 ^D^	298 ^D^	475 ^B^	248 ^BC^	36.6 ^C^	1340 ^A^
Acetic acid, 2-methylbutyl ester	877	885	508 ^A^	69.0 ^B^	65.9 ^B^	75.1 ^B^	414 ^A^	300 ^A^	221 ^A^	318 ^A^
Butanoic acid, propyl ester	896	898	4.73 ^A^	0.04 ^C^	0.02 ^C^	2.32 ^C^	3.86 ^B^	1.14 ^C^	0.13 ^D^	14.03 ^A^
Propanoic acid, butyl ester	906	908	56.1 ^A^	0.30 ^C^	0.03 ^C^	5.49 ^C^	40.2 ^A^	3.70 ^C^	0.77 ^D^	27.1 ^B^
Acetic acid, pentyl ester	911	915	93.0 ^A^	5.25 ^C^	0.35 ^D^	15.3 ^D^	80.5 ^A^	26.0 ^C^	0.89 ^D^	73.7 ^B^
2-Buten-1-ol, 3-methyl-, acetate	920	923	3.00 ^B^	4.94 ^A^	1.99 ^C^	0.73 ^C^	2.64 ^D^	22.9 ^A^	11.5 ^B^	4.56 ^C^
Hexanoic acid, methyl ester	923	923	n.d.	n.d.	n.d.	n.d.	0.68 ^C^	1.15 ^B^	1.09 ^B^	1.51 ^A^
Butanoic acid, 2-methyl-, propyl ester	945	938	5.02 ^A^	0.03 ^C^	n.d. ^C^	1.04 ^C^	3.02 ^B^	0.85 ^C^	0.02 ^D^	5.36 ^A^
Butanoic acid, butyl ester	993	996	22.7 ^A^	0.44 ^C^	0.01 ^C^	14.0 ^C^	21.1 ^B^	4.36 ^C^	0.27 ^D^	63.5 ^A^
Acetic acid, hexyl ester	1010	1013	230.9 ^A^	35.2 ^C^	1.97 ^D^	73.2 ^D^	95.4 ^B^	133 ^B^	15.5 ^C^	349 ^A^
Butanoic acid, 2-methylbutyl ester	1041	1041	55.9 ^A^	2.27 ^C^	0.13 ^C^	19.7 ^C^	27.7 ^B^	11.3 ^C^	1.57 ^D^	81.8 ^A^
Butanoic acid, 2-methyl-, 2-methylbutyl ester	1104	1104	11.5 ^A^	2.37 ^B^	0.33 ^D^	1.65 ^D^	3.06 ^B^	5.66 ^A^	0.37 ^C^	5.14 ^A^
Butanoic acid, hexyl ester	1191	1191	n.d. ^D^	0.66 ^C^	0.02 ^C^	7.98 ^B^	3.06 ^BC^	1.89 ^B^	0.39 ^B^	22.2 ^A^
Butanoic acid, 2-methyl-, hexyl est.	1237	1238	101 ^A^	4.74 ^B^	0.10 ^C^	19.2 ^C^	12.0 ^B^	3.99 ^C^	0.04 ^D^	36.2 ^A^
***Ketones***										
2-Butanone	599	600	23.9 ^A^	11.0 ^BC^	11.5 ^B^	9.00 ^B^	10.4 ^D^	46.2 ^B^	53.4 ^A^	35.7 ^C^
2-Pentanone	688	690	18.1 ^A^	5.05 ^B^	5.10 ^B^	4.06 ^B^	8.43 ^C^	16.0 ^AB^	20.5 ^A^	13.7 ^BC^
3-Pentanone	698	701	11.7 ^A^	3.92 ^B^	4.23 ^B^	2.46 ^B^	6.90 ^B^	17.3 ^A^	18.2 ^A^	7.4 ^B^
3-Pentanone, 2-methyl-	753	752	2.78 ^A^	0.56 ^B^	0.59 ^B^	0.43 ^B^	1.04 ^C^	1.86 ^B^	2.22 ^A^	1.23 ^C^
***Others***										
Butanoic acid, 2-methyl-	846	848	51.9 ^A^	0.73 ^B^	0.17 ^B^	0.87 ^B^	74.6 ^A^	30.6 ^B^	1.05 ^C^	7.56 ^C^
α-Pinene	946	944	n.d.	n.d.	n.d.	n.d.	0.20 ^A^	1.6 ^A^	1.02 ^A^	0.73 ^A^
Hexanal dimethyl acetal	977	980	n.d. ^D^	0.04 ^D^	0.02 ^D^	0.05 ^D^	2.63 ^C^	10.9 ^B^	8.39 ^B^	20.8 ^A^
Linalool oxide	1083	1088	n.d. ^D^	0.11 ^CD^	0.07 ^CD^	0.88 ^C^	0.92 ^B^	1.15 ^B^	0.59 ^C^	8.73 ^A^
Undecane	1100	1100	21 ^A^	4.26 ^B^	3.63 ^B^	5.02 ^B^	11.0 ^B^	22.9 ^A^	21.3 ^A^	27.6 ^A^
Dodecane	1203	1200	1.94 ^A^	0.41 ^B^	0.34 ^B^	0.55 ^B^	n.d.	n.d.	n.d.	n.d.
Tetradecane	1404	1400	0.20 ^A^	0.24 ^A^	0.18 ^A^	0.36 ^A^	n.d.	n.d.	n.d.	n.d.
β-Damascenone, (*E*)-	1406	1396	1.79 ^A^	0.52 ^B^	0.55 ^B^	0.48 ^B^	1.04 ^AB^	1.02 ^AB^	1.11 ^A^	0.49 ^B^

^a^ The identification was based on agreement between mass spectra of the analyzed compounds with those from MS libraries and LRI corresponding to those from databases (www.flavornet.org; www.odour.org.uk; Nist Webbook, accessed on 15 January 2023). The max. difference between RI from literature and our experiments is 10, due to possible differences in the experimental setup (inlet temp., temp.-ramp, column quality,…). Compounds with a match in the MS libraries but without reported linear retention indices in the databases were tentatively identified. ^b^ Linear temperature programmed retention index determined on a DB5 column. ^c^ Linear temperature programmed retention index from databases (www.flavornet.org; www.odour.org.uk; Nist Webbook, accessed on 15 January 2023). ^d^ ‘Relative concentration to the internal standard’ (mean, n = 2 or 3, resp.) collected from the headspace using HS-SPME calculated by comparison of the peak areas with that of the internal standard 2-octanol (25 ng absolute) with a response factor of 1. One-way ANOVA followed by post-hoc pairwise comparison using Tukey’s Honestly Significant Difference (HSD) test control (*p* < 0.05) to check for differences between single samples. ^t^ Compounds with a match in the MS libraries but without RI in the databases (n.a.) were tentatively identified. ^A,B,C,D^ Different superscript letters indicate statistically significant differences (*p* < 0.05) among the differently stored Crimson Crisp apples.

## Data Availability

This paper already contains all data available.
